# Intrinsic recreation of moderately uncertain events in macaques

**DOI:** 10.1016/j.isci.2026.115820

**Published:** 2026-04-18

**Authors:** Sakumi Iki, Haruhiko Iwaoki, Yuko Hattori, Ikuma Adachi

**Affiliations:** 1 The Hakubi Center for Advanced Research, Kyoto University, Kyoto, Japan; 2 Institute for the Evolutionary Origins of Human Behavior, Kyoto University, Aichi, Japan; 3 Advanced Neuroimaging Center, National Institutes for Quantum Science and Technology, Chiba, Japan

**Keywords:** behavioral neuroscience, cognitive neuroscience, psychology

## Abstract

Curiosity drives information-seeking without extrinsic incentives. Prior studies using looking-time measures suggest that curiosity peaks at intermediate uncertainty. Yet developmental and comparative research often reports that increased looking does not necessarily translate into action, leaving open whether animals intrinsically take overt actions generating intermediate uncertainty. Here, we show that monkeys are biased not only to look longer, but also to spontaneously interact with their environment in ways that recreate intermediate uncertainty. In a reward-free, hide-and-seek-like touchscreen game, Japanese macaques (*Macaca fuscata*) chose between two buttons that produced puppet appearances with different levels of spatial noise: intermediate vs. low (Experiment 1) and vs. high (Experiment 2). When noise variation was perceptible rather than imperceptible, macaques shifted their choice bias toward the intermediate-noise button by 9.6% (Experiment 1) and 13.5% (Experiment 2), and reselected it faster. Such intrinsic tendencies may optimize information gain by promoting engagement with stimuli offering maximal learning opportunities.

## Highlights

• A reward-free touchscreen game was developed for Japanese macaques
• Two buttons triggered puppet appearances with different noise levels
• Choice bias shifted toward selecting the intermediate-noise button
• The intermediate-noise button was reselected more quickly

## Graphical abstract

**Figure:**
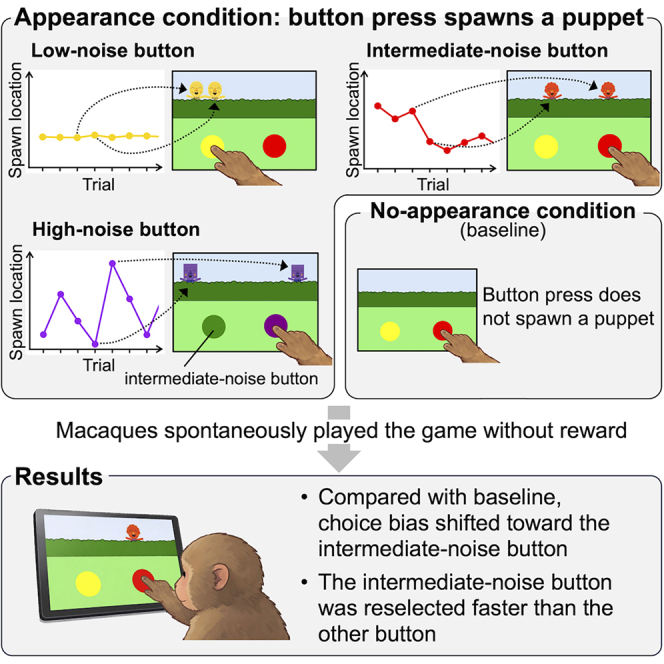

## Introduction

Humans and many other animals spontaneously explore their environments, not necessarily in pursuit of primary rewards such as food or mating opportunities (for reviews, see^1^^,^^2^^,^^3^^,^^4^^,^^5^). The intrinsic drive to seek information about the environment, independent of extrinsic incentives, is commonly termed curiosity.^6^^,^^7^^,^^8^^,^^9^ The motivational structure of curiosity and the properties of stimuli that most strongly evoke it have been debated for decades (e.g.,^3^^,^^10^). Recent studies support what is often referred to as the “Goldilocks principle” in the mechanisms of curiosity-driven information-seeking: Curiosity is biased toward stimuli of moderate complexity and uncertainty, rather than toward stimuli that are overly simple or excessively complex.^11^^,^^12^ For instance, human adults report the highest levels of epistemic curiosity in response to trivia questions associated with intermediate levels of uncertainty.^11^ Similarly, human infants^12^ and children^13^ show an attentional bias toward stimulus sequences of intermediate complexity, rather than toward those that are too simple or too complex. Although relatively few studies have examined whether this tendency extends to nonhuman animals, evidence from rhesus macaques (*Macaca mulatta*) suggests that they also show an attentional bias toward intermediately surprising information.^14^ Such a bias is thought to help optimize information gain by minimizing engagement with stimuli that are either unlearnably complex or effectively trivial.^2^^,^^3^^,^^13^

Because the studies cited above relied primarily on looking-time measures, the following point warrants attention. Prior research frequently finds that increased looking toward specific stimuli does not reliably predict subsequent overt exploration of those same stimuli. Indeed, counterintuitive “dissociations between seeing and acting” have often been reported (sensu^15^^,^^16^). For instance, while some studies show that human infants^17^^,^^18^ and dogs^19^ increase their physical engagement with objects involved in surprising events, another line of work has reported that, in both human children^20^ and adult nonhuman primates (e.g., rhesus macaques^15^; cotton-top tamarins, *Saguinus oedipus*^21^; and common marmosets, *Callithrix jacchus*^22^), increased looking time may indicate recognition that something unexpected has occurred, yet does not necessarily translate into appropriate search behavior. If an event possesses properties that an animal is intrinsically predisposed to find of interest, then not only attending to it but also interacting with the environment to elicit the event’s recurrence could be instrumental in securing additional opportunities to sample information. However, it remains unclear whether animals are biased not only to look longer but also to spontaneously interact with their environment in ways that recreate events of intermediate uncertainty and complexity (one related exception is a study showing that rats, *Rattus norvegicus*, prefer to play with partners of moderate novelty rather than with either highly familiar or completely novel partners^23^).

The distinction between the interaction-based bias examined in the present study and the attentional bias assessed in previous looking-time studies may appear trivial at first glance. Yet, it is deeply rooted in both classical and contemporary debates on exploratory behavior. According to Berlyne’s (1960)^10^ taxonomy, a foundational framework in curiosity research, exploratory behavior can be divided into three categories: (1) orienting responses, which involve changing the direction or state of the animal’s sense organs; (2) locomotor exploration, which involves moving the body through the environment; and (3) investigatory responses, which produce changes in objects within the environment through overt actions such as manipulation. Investigatory responses do not necessarily involve highly complex action sequences. In humans, for example, such responses can be as simple as pressing a switch to turn on a light in a dark room.^10^ Comparable examples observed in nonhuman animals, in both wild and experimental settings, include displacing a cover that conceals an object or opening a drawer and retrieving its contents.^10^ The present study is most closely related to the first and third categories. Directing gaze toward a particular stimulus constitutes a prototypical orienting response, whereas triggering an event by touching the touchscreen represents a canonical investigatory response.

To be precise, it may be misleading to characterize orienting responses as passive and investigatory responses as active forms of information-seeking. Both orienting and investigatory responses involve actively sampling task-relevant or ecologically relevant information. As such, they fall within the broader class of “active sensing” in contemporary cognitive science^24^ and can be viewed as lying along a common continuum. Based on Berlyne’s descriptions,^10^ the key difference between the two is that the former operates by effecting changes in the subject, whereas the latter operates by effecting changes in the environment. Put differently, an orienting response involves adjusting the animal’s own body (i.e., the sensory apparatus) to optimize information sampling, whereas an investigatory response involves changing the state of the environment to generate sensory data that would otherwise be unobtainable. In this sense, the latter can be viewed as an extension of active sensing toward what contemporary cognitive science and robotics call “interactive perception” (^25^^,^^26^; see also a recent neuroscience framework^27^ that distinguishes “alloactive” sensing, in which an animal moves only its sensors, from “homeoactive” sensing, in which it changes the state of the environment). Accordingly, investigatory responses (or interactive perception) may recruit higher-level neural and cognitive mechanisms beyond those implicated in prototypical active sensing, such as gaze orienting. These may include encoding associative, and perhaps causal, relationships between one’s own actions and ensuing environmental changes, as well as predictive processes concerning the consequences of these interactions.^25^^,^^26^ By taking into account both this continuity and potential mechanistic and functional differences, examining not only the attentional biases that have been studied in prior work^14^^,^^15^^,^^16^ but also interaction-based biases in nonhuman animals is expected to enrich a phylogenetically informed understanding of curiosity-driven information-seeking.

**Figure 1:**
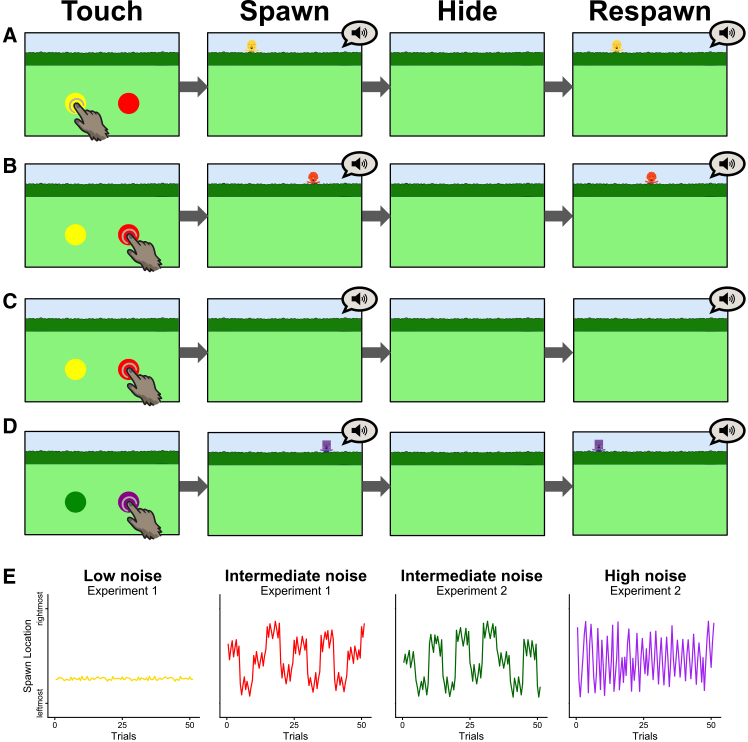
Overview of Experiments 1 and 2 (A, B, and D) In the appearance condition, when a subject pressed a button, a puppet of the same color spawned from behind the hedge at a location sampled from the corresponding probabilistic parameter, crouched to hide, respawned from another parameter-determined location, and hid again. A bell tone played at each appearance. Panels show examples of (A) the low-noise and (B) intermediate-noise spawn sequences in Experiment 1, and (D) the high-noise spawn sequence in Experiment 2. (C) In the no-appearance condition, no puppet was displayed and only bell tones were presented. (E) Example spawn locations across trials within a session for each button type. Each session included up to 102 spawn events (two spawns per button press × 51 trials).

Building on the above discussion, we investigate whether perceiving variation in uncertainty intrinsically shapes investigatory, interaction-based responses in adult Japanese macaques (*M. fuscata*) in a touchscreen game. We adapt a simplified version of the “hide-and-seek” touchscreen game, originally developed for human children^28^ and adults,^29^ for use with nonhuman primates. To focus on intrinsic information-seeking biases, the task provides no external rewards. On each trial, two on-screen buttons appear, and the subject selects one. Upon selection, a puppet emerges from behind a hedge at a location sampled from a specified probabilistic distribution, then crouches, disappears, and reappears at another location resampled from the same distribution before hiding again (Figures 1A, 1B, and 1D; Video S1). This spawn-respawn sequence is implemented to make the variability in spawn location apparent within a single trial. In Experiment 1, one button (“low-noise”) generates puppet locations with minimal variance (0.03) around a fixed mean (Figures 1A and 1E), whereas the other (“intermediate-noise”) generates locations with greater variance (0.15) and a mean that shifts every five trials of that button (Figures 1B and 1E). In Experiment 2, the “intermediate-noise” button, presented in a different color and triggering a different puppet than in Experiment 1, is paired with a “high-noise” button whose puppet locations are randomly generated without a stable mean, resulting in a variance of 0.45 (Figures 1D and 1E). Across both experiments, we assess which button type the macaques select in the absence of extrinsic incentives. To ensure that subjects experience both contingencies and can form expectations about each, we intermittently include forced-choice trials during which only one button is displayed.

Video S1. Demonstration of the touchscreen-based hide-and-seek-like game played by a human experimenter, related to STAR Methods In this example, the yellow button is associated with low spatial noise, and the red button with intermediate spatial noise in the puppet’s spawn location. The first two trials are forced-choice trials, followed by repeated cycles of ten optional-choice and two forced-choice trials.

As is common in two-choice tasks with nonhuman animals, inherent color and side biases can shift nominal “chance-level” responding away from 50%. Indeed, our subjects exhibited significant color and side biases in both Experiments 1 and 2 (see results). Under these circumstances, treating 50% as a fixed chance benchmark and testing for deviations from it does not cleanly isolate differences in choice probability between the two options that are specifically attributable to choice-contingent event uncertainty. To account for such pre-existing color/side biases and avoid this problem, we implement a “no-appearance” condition as a baseline (Figure 1C). In this condition, the timing of the auditory feedback following a button press is identical to that in the experimental (“appearance”) condition, but the puppet never appears. Each subject completes both the appearance and no-appearance conditions twice in alternating order. We then quantify the shift in button-choice bias from the no-appearance condition to the appearance condition, thereby estimating the extent to which variability in puppet-appearance locations influences macaques’ intrinsic choice behavior.

In this work, using the task design described above, we show that even in the absence of external rewards, macaques exhibit an intrinsic bias toward interacting with their environment in ways that generate repeated occurrences of events characterized by intermediate levels of uncertainty and complexity. In both Experiments 1 and 2, we analyze two key behavioral measures: (1) which button subjects select (i.e., binary choice) and (2) the latency to reselect the same button as on the immediately preceding trial (i.e., response time). Across both experiments, we observe a bias shift toward selecting the intermediate-noise button in the appearance condition, where differences in noise level are perceptible, compared with the no-appearance baseline condition, where choice is driven primarily by pre-existing color/side biases. Furthermore, our results suggest that exposure to stimuli of intermediate uncertainty elicits greater motivational vigor, leading subjects to revisit them more promptly. Accordingly, under the appearance condition, the intermediate-noise button is reselected more quickly on the next trial than the low-noise button (in Experiment 1) or the high-noise button (in Experiment 2), whereas under the no-appearance condition, response times do not differ significantly across button types. Taken together, these findings build on prior gaze-based work on curiosity by showing that perceiving variation in uncertainty shapes not only attentional orienting, as demonstrated in prior work,^12^^,^^13^^,^^14^ but also investigatory, interaction-based responses that effect changes in the environment to re-evoke intermediate uncertainty.

## Results

### Experiment 1

First, to ensure we analyzed data from periods when subjects remained continuously engaged with the game, we estimated the cutoff time *τ* using survival analysis. We defined τ as the smallest interval at which the probability of the occurrence of the next button press satisfied *S*(τ) ≤ 0.05. If a subject made no button presses for a continuous interval of at least *τ*, we considered the subject to have lost interest. We truncated the session at that point, excluding any subsequent button presses from analysis. Based on the survival analysis, *τ* was estimated to be 174.38 s (Figure S1). Using this value as the cutoff, we applied the following inclusion criteria for the main analyses: subjects had to (1) complete at least five optional-choice trials within a single session and (2) meet this criterion in at least two sessions. As a result, 8 of 11 subjects met the inclusion criteria.

**Figure 2:**
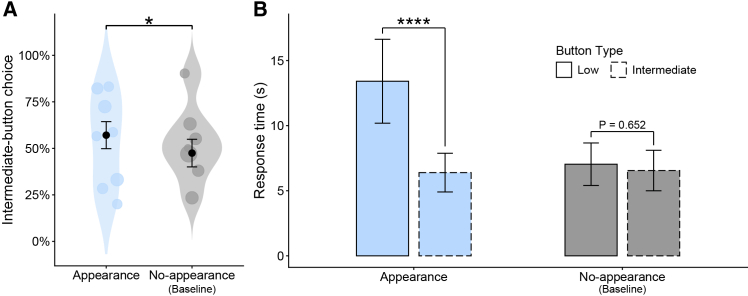
Results of Experiment 1 (A) Predicted probability of choosing the intermediate-noise button from the binomial GLMM. Points show subject means; point size is proportional to the number of observations. Sample size: *N* = 870 button choices. ∗*p* < 0.05 (GLMM). (B) Predicted response time from the gamma GLMM for trials in which subjects consecutively selected the same button as on the immediately preceding trial. Sample size: *N* = 512 consecutive same-button selections. Bars with solid outlines, low-noise; bars with dashed outlines, intermediate-noise. ∗∗∗∗*p* < 0.0001 (Tukey’s post hoc comparison). In both panels, blue indicates the appearance condition and gray indicates the baseline no-appearance condition. Error bars represent standard errors.

We tested for condition-dependent shifts in bias toward choosing the intermediate-noise button using a binomial generalized linear mixed model (GLMM; see STAR Methods; raw choice proportions prior to controlling for covariates are provided in Table S1). The full model, which included condition (appearance vs. no-appearance) as the key predictor alongside control variables, fit the data significantly better than the null model containing only the control variables (likelihood ratio test: χ^2^ = 5.51, df = 1, *p* = 0.0189; Table S2). In the appearance condition, in which the puppet was displayed on the screen, subjects were significantly more likely to choose the intermediate-noise button than in the baseline no-appearance condition (GLMM: condition [no-appearance], β = −0.387 ± 0.166, *p* = 0.019; Figure 2A; Table S3), consistent with our prediction. Holding the continuous covariates at their sample means and the categorical covariates at their observed proportions, the predicted probability of selecting the intermediate-noise button was 57.1% in the appearance condition and 47.5% in the no-appearance condition, indicating a statistically significant 9.6% bias shift. In addition, the type of the last button pressed had a significant effect, indicating a higher probability of repeating the same button on consecutive trials (GLMM: last button type [low-noise], β = −0.312 ± 0.152, *p* = 0.040; Table S3). Because trial number and session number showed no significant effects (GLMM: trial number, β = 0.009 ± 0.005, *p* = 0.060; session number, β = 0.028 ± 0.075, *p* = 0.709; Table S3), we further assessed possible learning effects by fitting a supplementary GLMM restricted to two sub-blocks within the appearance condition: the first five optional-choice trials of the first session and the last five optional-choice trials of the second session. The probability of selecting the intermediate-noise button did not differ between sub-blocks (GLMM: sub-block [last], β = −0.530 ± 0.487, *p* = 0.277; Table S4). Moreover, using only data from the no-appearance condition, in which no puppet appeared, we examined whether button choices exhibited inherent color and side biases. Subjects were significantly more likely to press the red/right button than the yellow/left button (GLMM: color/side [yellow/left], β = −1.392 ± 0.425, *p* = 0.001; Table S5). This confirms the presence of pre-existing perceptual biases in button choice, indicating that a 50% choice probability cannot be treated as a fixed chance benchmark and thereby justifying our analytic approach of testing bias shifts by comparing the baseline and experimental conditions. To quantify the tendency within the experimental condition alone, we fitted a supplementary GLMM using only data from the appearance condition (Table S6). After adjusting for color/side biases as well as the other control variables, the estimated probability of selecting the intermediate-noise button was 57.3% (95% CI: 44.8%, 68.9%). This indicates that although a significant bias shift can be detected when comparing the appearance condition with the baseline no-appearance condition, the magnitude of this bias is not large enough to be detected when testing within the appearance condition alone.

In addition, we analyzed response time in trials where subjects consecutively selected the same button, defined as the latency from button presentation to the subsequent press on the second trial. We fitted a gamma GLMM with a log link (see STAR Methods). The full model, which included condition (appearance vs. no-appearance), button type (intermediate-noise vs. low-noise), and their interaction as the key predictors, together with control variables, fit the data significantly better than the null model containing only the control variables (likelihood ratio test: χ^2^ = 21.90, df = 3, *p* < 0.0001; Table S2). Under the appearance condition, subjects responded significantly faster when repeatedly choosing the intermediate-noise button than when repeatedly choosing the low-noise button (Tukey’s post hoc test: intermediate-noise vs. low-noise in the appearance condition, ratio = 0.477 ± 0.085, *p* < 0.0001; Figure 2B; Table S8). In contrast, no significant differences in response times across button types were observed under the no-appearance condition (intermediate-noise vs. low-noise in the no-appearance condition, ratio = 0.931 ± 0.147, *p* = 0.652; Figure 2B; Table S8), consistent with our prediction. Taken together, in contrast to choice probability, these results reveal significant button-type-dependent differences in response time that are reliably detectable even within the experimental condition alone. Moreover, both trial number and session number had significant effects: later trials and later sessions were associated with slower response times on the second press of consecutive same-button selections (GLMM: trial number, β = 0.024 ± 0.004, *p* < 0.0001; session number, β = 0.367 ± 0.059, *p* < 0.0001; Table S7; Figures S2A and S2B). This pattern may indicate that subjects’ motivation to engage with the game declined over time.

### Experiment 2

Based on the survival analysis, *τ* was estimated to be 931.30 s (Figure S3). We used this value as the cutoff. Consequently, five individuals met the inclusion criteria for the main analyses.

**Figure 3:**
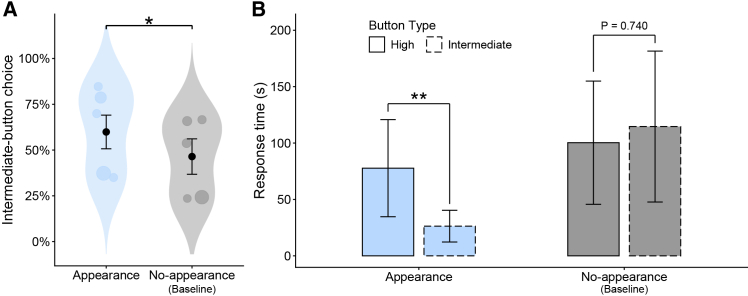
Results of Experiment 2 (A) Predicted probability of choosing the intermediate-noise button from the binomial GLMM. Points show subject means; point size is proportional to the number of observations. Sample size: *N* = 404 button choices. ∗*p* < 0.05 (GLMM). (B) Predicted response time from the gamma GLMM for trials in which subjects consecutively selected the same button as on the immediately preceding trial. Sample size: *N* = 251 consecutive same-button selections. Bars with solid outlines, high-noise; bars with dashed outlines, intermediate-noise. ∗∗*p* < 0.01 (Tukey’s post hoc comparison). In both panels, blue indicates the appearance condition and gray indicates the baseline no-appearance condition. Error bars represent standard errors.

To test for condition-dependent bias shifts toward choosing the intermediate-noise button, we fitted a binomial GLMM (see STAR Methods; raw choice proportions prior to controlling for covariates are provided in Table S1). The full model, which included condition (appearance vs. no-appearance) as the key predictor together with control variables, explained significantly more variance than the null model containing only the control variables (likelihood ratio test: χ^2^ = 5.09, df = 1, *p* = 0.0241; Table S2). In the appearance condition, where the puppet was shown on the screen, subjects were more likely to choose the intermediate-noise button than in the baseline no-appearance condition (GLMM: condition [no-appearance], β = −0.543 ± 0.242, *p* = 0.0245; Figure 3A; Table S9), consistent with our prediction. Holding the continuous covariates at their sample means and the categorical covariates at their observed proportions, the predicted probability of selecting the intermediate-noise button was 59.9% in the appearance condition and 46.4% in the no-appearance condition, indicating a statistically significant 13.5% bias shift. As in Experiment 1, because trial number and session number showed no significant effects (GLMM: trial number, β = −0.016 ± 0.010, *p* = 0.0985; session number, β = 0.166 ± 0.106, *p* = 0.1183; Table S9), we further assessed possible learning effects by fitting a supplementary GLMM restricted to the first and last five optional-choice trials within the appearance condition. Again, the probability of selecting the intermediate-noise button did not differ between the first and last five trials (GLMM: sub-block [last], β = −0.142 ± 0.666, *p* = 0.831; Table S10). We also examined, using only data from the no-appearance condition, whether button choices exhibited inherent color and side biases. Subjects were significantly more likely to press the purple/right button than the green/left button (GLMM: color/side [purple/right], β = 0.906 ± 0.457, *p* = 0.048; Table S11). This confirms the presence of pre-existing perceptual biases in button choice and indicates that a 50% choice probability cannot be treated as a fixed chance benchmark. To quantify the tendency specifically within the experimental condition, we fitted a supplementary GLMM using only data from the appearance condition (Table S12). After adjusting for color/side biases as well as the other control variables, the estimated probability of selecting the intermediate-noise button was 58.2% (95% CI: 40.6%, 74.0%). As in Experiment 1, this pattern indicates that although a significant bias shift can be detected when comparing the appearance condition with the baseline no-appearance condition, the magnitude of this bias is not large enough to be reliably detected when testing within the appearance condition alone.

For response time in trials where subjects consecutively selected the same button, we fitted a gamma GLMM with a log link (see STAR Methods). The full model, which included condition (appearance vs. no-appearance), button type (intermediate-noise vs. high-noise), and their interaction as the key predictors, together with control variables, fit the data significantly better than the null model that contained only the control variables (likelihood ratio test: χ^2^ = 22.62, df = 3, *p* < 0.0001; Table S2). Under the appearance condition, responses were significantly faster when repeatedly selecting the intermediate-noise button than when repeatedly selecting the high-noise button (Tukey’s post hoc test: high-noise vs. intermediate-noise in the appearance condition, ratio = 2.952 ± 0.971, *p* = 0.001; Figure 3B; Table S14). In contrast, under the no-appearance condition, response time did not differ significantly between button types (high-noise vs. intermediate-noise in the no-appearance condition, ratio = 0.875 ± 0.352, *p* = 0.740; Figure 3B; Table S14), consistent with our prediction. In contrast to choice probability, as in Experiment 1, these results reveal significant button-type-dependent differences in response time that are reliably detectable within the experimental condition alone. We also observed significant positive effects of trial number and session number on latency, such that later trials and later sessions were associated with slower second-press latencies when repeating the same button, which may indicate declining motivation to engage with the game over time (GLMM: trial number, β = 0.056 ± 0.010, *p* < 0.0001; session number, β = 0.541 ± 0.110, *p* < 0.0001; Table S13; Figures S4A and S4B).

## Discussion

Overall, our findings suggest that in primates, the perception of variation in levels of uncertainty intrinsically influences not only attentional allocation in orienting responses, as previously shown,^14^ but also investigatory responses (or interactive perception) that effect changes in the environment. Across both experiments, under the appearance condition, where a puppet appeared at locations sampled from a specified probability distribution, subjects were significantly more likely to select the intermediate-noise button than under the baseline no-appearance condition, in which no puppet appeared. This bias shift, observed after controlling for inherent color and side biases, indicates a selective increase relative to baseline in interactions that generate events characterized by moderate levels of uncertainty. In addition, under the appearance condition, subjects reselected the intermediate-noise button more quickly on the subsequent trial than they did the low- or high-noise buttons. By contrast, under the no-appearance condition, response times for successive selections did not differ significantly across button types. Taken together, the observed condition-dependent shifts in choice bias and reductions in reselection latency indicate that exposure to intermediately uncertain events intrinsically biases macaques to interact with their environment in ways that recreate such events, thereby presumably allowing them to acquire additional information about them.

The absence of effects of trial number or session number suggests that there were no significant learning-related changes in button choice. By contrast, response times on repeated selections of the same button increased across trials and sessions, which may indicate a decline in motivation to re-engage quickly with the game. In addition, a considerable proportion of individuals failed to meet the minimum inclusion criteria (≥2 sessions with ≥5 trials; Experiment 1: one male and two females; Experiment 2: two males and four females, including the same three from Experiment 1 who did not qualify). This pattern may reflect rapid disengagement or boredom with a task that provided no external rewards. Indeed, this tendency was more pronounced in Experiment 2, which was conducted after exposure to Experiment 1: button selections were more widely spaced in time, response times were substantially longer than in Experiment 1 (Figures 2B and 3B), and, correspondingly, the cutoff threshold estimated by the survival analysis was greater (Figures S1 and S3). However, these observations do not necessarily imply that our captive subjects were particularly unmotivated to engage with the task. For instance, in the hide-and-seek game used by Poli et al.,^28^ a task that was more complex than ours, as it required human children to explicitly infer the puppet’s hidden location behind a hedge, the average number of completed trials was only 27 (note that a direct comparison is not possible because, in their study, sessions ended once a child touched any of the buttons 35 times). Similarly, Pelz and Kidd^30^ reported that children under 22 months of age never interacted with a touchscreen-based game for more than 5 min. These studies imply that even human children, who, according to the comparative literature, exhibit stronger curiosity than other great apes,^31^ rarely sustain intrinsically motivated exploration for extended periods. Thus, our subjects should not necessarily be considered particularly unmotivated to engage with the game task. At the same time, the fact that the same three individuals failed to meet the inclusion criteria in both Experiments 1 and 2 suggests that their lack of engagement may reflect a stable behavioral phenotype. Such individual differences may reflect personality traits or behavioral syndromes, such as “boldness” or “exploration,” that have been identified in other primate studies using different measures.^32^^,^^33^^,^^34^

In conclusion, our results demonstrate that when variation in uncertainty is perceptible, compared with when it is not, macaques exhibit a bias shift toward interactions that generate events of intermediate uncertainty. Such a bias may carry adaptive value in the wild by limiting unnecessary interactions with stimuli that are either effectively unlearnable (too complex) or trivial (too simple), while promoting engagement with stimuli most likely to afford future learning opportunities.^3^^,^^4^ Our findings build on previous gaze-based research by demonstrating that macaques are biased not only to look longer at intermediately uncertain events, as previously reported,^14^ but also to interact with their environment in ways that recreate such events. Given that prior developmental and comparative research has shown that increased looking does not necessarily translate into overt actions, our results provide novel insights into the perceptual-behavioral link in the context of curiosity-driven information-seeking. Future work aimed at identifying the neural and cognitive mechanisms linking the perception of intermediate uncertainty to overt, interaction-based information-seeking behavior will contribute to a more comprehensive understanding of this phenomenon.

### Limitations of the study

When generalizing the present findings, several caveats should be kept in mind. First, although response-time changes were clear and statistically significant even within the experimental appearance condition alone, the bias shift in choice probability was detectable only relative to the baseline condition and, although statistically significant, was modest in magnitude. This suggests that, given our task design, the magnitude of behavioral change induced by perceiving differences in uncertainty level should not be overestimated. That said, discrepancies in the magnitude of behavioral biases indexed by choice behavior versus response time are common in comparative cognition research across a variety of paradigms (e.g., in hens, cognitive judgment bias tests reveal stronger biases when latency is used as the index than when choice is used^35^; in macaques, significant “pessimistic” judgment biases in a go/no-go visual discrimination task emerged only when response time was indexed in addition to go/no-go choice rates^36^^,^^37^; and in a speeded visual classification paradigm, auditory interference from task-irrelevant distractor tones manifested primarily as slower responses in humans but as higher rates of choice errors in chimpanzees^38^). It has been argued that whether behavioral and cognitive biases are expressed more strongly in response time or in choice rate may reflect species- and individual-level variation in relevant factors, such as hesitation, impulsivity, and inhibitory control.^38^ In light of these considerations, the fact that only response speed, and not choice rate, revealed a bias that was detectable within the experimental condition alone does not necessarily imply that our effects are fragile. Rather, our findings provide a basis for future work examining how temperament and personality traits in our study species shape intrinsic exploratory behavior.

Second, our study involved a limited number of subjects, and future research with larger sample sizes will be necessary to draw more robust conclusions. Relatedly, all of our subjects were adults, which prevented us from examining potential developmental influences. In general, immature mammals tend to exhibit stronger curiosity and greater playfulness than adults, as they typically remain under parental care, are to some extent exempt from inter-individual competition, and are still in the process of acquiring a wide range of skills and knowledge (e.g.,^32^^,^^39^). Once they reach sexual maturity and become integrated into the group’s dominance hierarchy, these tendencies often diminish. Thus, it is possible that juvenile individuals may be more intrinsically motivated to engage with tasks such as the present one and may also be more attracted to noisier stimuli than adults.

Third, this study was conducted with captive animals. Captive individuals typically experience a chronic reduction in environmental stimulation compared to their wild counterparts,^40^^,^^41^^,^^42^ which may increase their motivation to take actions that generate even minimally varying stimuli and shift the thresholds for perceiving low, intermediate, or high levels of complexity. Although controlled game-like tasks such as the present one are difficult to implement in the wild, investigating whether responses to stimulus complexity or uncertainty vary depending on living conditions remains a promising direction.

Fourth, since this study focused solely on variation in spatial noise, it remains unclear whether our findings can be generalized to other modalities (e.g.,^43^^,^^44^) or forms of stimulus complexity. For instance, it would be valuable to investigate active information-seeking in response to temporal variation or to violations of physical expectations, with unpredictability systematically manipulated. Furthermore, although our stimuli were unrelated to reward, future research should also test how unexpected reward-related events, such as surprising increases in, or omissions of, an expected food reward (e.g.,^45^), influence curiosity-driven information-seeking actions.

Finally, given that both the intensity and type of curiosity and playfulness are known to vary across species (see review in^4^), caution is warranted when generalizing the findings of this study to other taxa. For example, even among primates, generalist species with opportunistic foraging strategies, such as chacma baboons (*Papio ursinus*), tend to display greater curiosity toward novel objects than herbivorous specialists, such as gelada baboons (*Theropithecus gelada*).^46^ Among great apes, highly social species such as bonobos (*Pan paniscus*) and chimpanzees (*Pan troglodytes*) may exhibit stronger suppression of curiosity when tested in isolation, compared to more solitary species such as Sumatran orangutans (*Pongo abelii*) and Bornean orangutans (*Pongo pygmaeus*).^47^ More generally, species occupying distinct socioecological niches are expected to respond differently to stimuli that vary in complexity and ecological relevance.

## Resource availability

### Lead contact

Further information and requests should be directed to and will be fulfilled by the lead contact, Sakumi Iki (sakumi.iki@gmail.com).

### Materials availability

This study did not generate new, unique reagents.

### Data and code availability

All data and code have been deposited on OSF: https://osf.io/24b6u/overview. The data and code are publicly available. Any additional information required to reanalyze the data reported in this paper is available from the lead contact upon request.

## Acknowledgments

This study was supported by 10.13039/501100001691JSPS 10.13039/501100001691KAKENHI grant number JP25K21531 and by the Hakubi project at 10.13039/501100005683Kyoto University. We are grateful to Dr. Poli for kindly granting permission to modify and use the original program and illustrations.

## Author contributions

S.I., H.I., Y.H., and I.A. conceived the study and finalized the manuscript. S.I. acquired the funding, gathered the data, performed the statistical analyses, and drafted the manuscript.

## Declaration of interests

The authors declare no competing interests.

## STAR★Methods

### Key resources table

**Table:** 

REAGENT or RESOURCE	SOURCE	IDENTIFIER
**Deposited data**		

Raw data and R code	This paper	https://osf.io/24b6u/overview

**Experimental models: Organisms/strains**		

Japanese macaques (*Macaca fuscata*)	Center for the Evolutionary Origins of Human Behavior, Japan	N/A

**Software and algorithms**		

R	R Foundation	https://www.r-project.org/

### Experimental model and study participant details

Eleven adult Japanese macaques housed at the Center for the Evolutionary Origins of Human Behavior (EHUB), Kyoto University, participated in the study. The subjects were three males and eight females, aged 10–20 years (mean ± SD = 15.7 ± 3.2 years; Japanese macaques typically reach sexual maturity at approximately 4 years of age). Animals were housed individually or in pairs; for paired housing, an opaque partition was inserted at least 30 min before testing to separate them. To focus on intrinsic information-seeking, no responses in the task were reinforced with extrinsic rewards (e.g., food). Two to three hours after completing each day’s session, subjects were fed their daily ration. Water was available *ad libitum* throughout the study. All procedures were approved by the Animal Welfare and Care Committee of EHUB, Kyoto University (#2025-105), complied with the Japanese Act on the Welfare and Management of Animals, and are reported in accordance with the ARRIVE guidelines.

### Method details

#### Apparatus

The game task was conducted on a touchscreen tablet (Microsoft Surface Pro 8 EIV-00010) running a program written in Microsoft Visual Basic 2010. Subjects’ button choices and response times were recorded using the touchscreen. Sessions were video-recorded using GoPro HERO11 and HERO12 cameras positioned above the touchscreen and the cage.

#### Experimental procedure

##### Experiment 1

Experiment 1 was conducted from September to October 2025 on weekdays. On the day before testing began, subjects were habituated for 20 min to a screen that displayed only a lawn and a hedge, with no buttons present. Testing took place in a blocked design, where appearance and no-appearance (baseline) conditions alternated daily. Each subject completed two sessions per condition. Condition order was counterbalanced across subjects in one of two sequences: appearance → no-appearance → appearance → no-appearance, or no-appearance → appearance → no-appearance → appearance. The task was a modification of the hide-and-seek computer game developed for human adults and children.^28^^,^^29^ The mapping between button color and spawn-location parameter was counterbalanced across subjects and then held constant within each subject across the four sessions. This design was intended to avoid a potential confound that would arise if the mapping were changed within subjects across sessions, because subjects would then need to relearn the mapping at the start of each session. For the same reason, the combination of button color and on-screen position was also kept constant: the yellow button always appeared on the left, and the red button always appeared on the right.

Each session began with a 2-min start screen. The display then switched to an illustration of a hedge and lawn, after which the following procedure commenced immediately.

1. **Two forced-choice trials.** A single button, either yellow or red, associated with the low-noise parameter (see below), appeared at the bottom of the screen. In the appearance condition, pressing the button caused a puppet of the same color to spawn from behind the hedge at a location sampled from that parameter, crouch to hide, and then respawn from another location resampled from the same parameter before hiding again. This spawn–respawn sequence was implemented so that even a single button press exposed subjects to variability in the puppet’s spawn location. A bell tone was played through the Surface computer’s speakers at each spawn event. The elapsed time from the puppet’s first appearance to its hiding after the respawn was 3.4 s. Immediately after the puppet hid following the respawn, only the other color button, which was associated with the intermediate-noise parameter, was presented. We presented the low-noise forced-choice trial first so that the higher-noise trial always followed the lower-noise trial, thereby enhancing the salience of the higher relative noise. When the subject pressed this second button, a puppet matching the button’s color spawned at locations determined by the intermediate-noise parameter, hid, respawned, and hid again. In the no-appearance condition, the timing of the two bell tones after a press and the timing of the presentation of the next-trial button were identical to those in the appearance condition, but no puppet was displayed.
2. **Ten optional-choice trials.** Red and yellow buttons were displayed side by side on the lawn. Each time a button was pressed, the corresponding puppet executed the same spawn–hide–respawn–hide sequence as in Step 1, according to that button’s parameter. In the no-appearance condition, no puppet was displayed; however, the timing of auditory feedback and the subsequent button presentation matched that of the appearance condition.
3. **Two additional forced-choice trials.** The procedure was the same as in Step 1, with the order of the two buttons alternating between intermediate-noise → low-noise and low-noise → intermediate-noise.
4. **Repetition and stopping rule.** Steps 2 and 3 were repeated until one of the two buttons had been pressed a total of 51 times (including the forced-choice trials in Step 1) or until 30 min had elapsed from the start of Step 1, whichever occurred first; the session then ended. This procedure, in which the session terminated once either button had been pressed a predetermined number of times, followed previous work.^28^^,^^29^

##### Experiment 2

Experiment 2 was conducted in October 2025 on weekdays. As in Experiment 1, the appearance and no-appearance conditions alternated, with each subject completing two sessions per condition. The procedures matched Experiment 1 except in two respects: (1) the buttons were green and purple rather than red and yellow, to avoid any bias arising from differential novelty between the buttons, because keeping the intermediate-noise button the same color as in Experiment 1 would make it less novel than the other option; and (2) the probabilistic parameters determining puppet locations were changed such that the intermediate-noise button was paired with a high-noise button, rather than with a low-noise button. The green button always appeared on the left, and the purple button always appeared on the right. In the Step 1 forced-choice trials, the button associated with the intermediate-noise parameter was presented first, followed by the high-noise button. The intermediate-noise parameters in Experiment 2 were identical to those in Experiment 1 in variance and in the schedule of mean shifts, although the actual location values were newly sampled.

As in Experiment 1, Steps 2 and 3 were then repeated until one of the two buttons had been pressed a total of 51 times, or until 30 min had elapsed from the start of Step 1, whichever came first; the session then ended.

#### Stimuli

Although presenting a realistic, macaque-like character could help capture the animals’ attention effectively,^48^ displaying unfamiliar conspecifics could also introduce additional confounds, such as fear responses. Therefore, we modified the cartoon-like puppet stimulus used in prior studies^28^^,^^29^ and used it as the visual stimulus that spawned each time a button was pressed. Spawn locations were defined along a one-dimensional axis spanning the screen width from −0.9 (left edge) to 0.9 (right edge) and were generated pseudorandomly by simulation. For the low-noise sequence, we generated 102 locations (two spawns per button press × 51 trials) by sampling from a normal distribution with a randomly selected mean and very small variance (0.03). For the intermediate-noise sequence, we again generated 102 locations from a normal distribution with variance 0.15 around a mean that shifted at predetermined intervals to retain partial predictability while introducing uncertainty: after the first 9 points, the mean shifted every 10 points for nine blocks, followed by a final block of 3 points (9 + 90 + 3 = 102). To prevent points from ending up very close together by chance within a block, we enforced a minimum separation of 0.08 units between consecutive samples. For the high-noise sequence, which lacked a stable mean, we sampled 102 locations from a continuous uniform distribution on [−0.9, 0.9]. To prevent points from ending up very close together by chance, we enforced a minimum separation of 0.25 units between consecutive samples; the resulting series had a sample variance of approximately 0.45. Example spawn sequences are shown in Figure 1.

### Quantification and statistical analysis

To exclude outlier button presses occurring after prolonged inactivity, we estimated a no-press threshold τ using a Kaplan–Meier survival analysis. We analyzed the data in R (version 4.5.1) with the “survival” package (function *survfit*). For this analysis, we examined Experiments 1 and 2 separately, including only sessions where subjects pressed the button at least twice and thus completed the initial forced-choice phase. The response variable was the inter-press interval (time from one press to the next). Intervals were right-censored at 30 min from the session start. We set α = 0.05 and defined τ as the smallest latency such that the probability of a subsequent press occurring after that time was ≤ α.

For the subsequent analyses of binary choice and response time, we fitted GLMMs using the *glmer* function in the “lme4” package (α = 0.05). We truncated each session at the first no-press interval ≥ τ and excluded all subsequent events. We included only sessions with at least five optional-choice trials and removed individuals who did not meet this session-level criterion in at least two sessions.

We then analyzed button choice in the optional-choice trials using a binomial GLMM with a logit link. Experiments 1 and 2 were modeled separately. The response variable was a binary indicator of choosing the “intermediate-noise” button (coded as 1) versus the comparison button (coded as 0: the “low-noise” button in Experiment 1 and the “high-noise” button in Experiment 2). The key predictor was condition (categorical: appearance vs. no-appearance). Control variables were session number (continuous, 1–4), optional-choice trial number within session (continuous), the button chosen on the immediately preceding trial (categorical: low vs. intermediate in Experiment 1; high vs. intermediate in Experiment 2), and subject age (continuous). In the Experiment 1 model, sex was included as a fixed effect; in the Experiment 2 model, sex was not included because only one male met the inclusion criterion of completing at least two sessions with at least five optional-choice trials, which precluded reliable estimation of a separate sex effect. To address potential pseudoreplication, we included subject ID as a random intercept. We assessed the significance of the full model by comparing it with a null model that contained only the control variables and the random intercept, using a likelihood-ratio test via the *anova* function. To provide a descriptive index of the magnitude of the condition-dependent bias shift, we used the *emmeans* function in the “emmeans” package to compute the predicted probability of selecting the intermediate-noise button in each condition, holding continuous covariates at their sample means and categorical covariates at their observed proportions. We also assessed potential learning effects by fitting a supplementary GLMM restricted to two sub-blocks within the appearance condition: the first five optional-choice trials of the first session and the last five optional-choice trials of the second session. This model included the following fixed effects: sub-block (categorical: first vs. last), the button chosen on the immediately preceding trial (categorical: low vs. intermediate in Experiment 1; high vs. intermediate in Experiment 2), and subject age (continuous). As in the main model, sex was included as a fixed effect in Experiment 1 but not in Experiment 2.

Furthermore, using data from the no-appearance condition only, we examined whether button choices exhibited inherent color or side biases. As in the main analyses, the response variable was button type, coded as 1 if the pressed button corresponded to the one associated with the intermediate-noise parameter in the appearance condition for that subject, and 0 otherwise. Fixed effects in the model were the color/side of the button associated with the intermediate-noise parameter (categorical: yellow/left vs. red/right in Experiment 1; green/left vs. purple/right in Experiment 2), session number (continuous), optional-choice trial number within session (continuous), the button chosen on the immediately preceding trial (categorical: yellow/left vs. red/right in Experiment 1; green/left vs. purple/right in Experiment 2), and subject age (continuous). Sex was included as a fixed effect in Experiment 1 but not in Experiment 2. To quantify the tendency specifically within the experimental condition, we fitted a supplementary GLMM using only data from the appearance condition. The response variable and fixed effects in this model were identical to those used in the analysis of the no-appearance condition described above. The estimated probability of selecting the intermediate-noise button, adjusted for the effects of all predictors, was obtained using the *emmeans* function, holding continuous covariates at their sample means and categorical covariates at their observed proportions.

We used a gamma GLMM with a log link to test whether, in optional-choice trials, subjects responded faster when repeatedly choosing the intermediate-noise button than when repeatedly choosing the comparison button. As in the choice analyses, Experiments 1 and 2 were analyzed separately. The response variable was the response time on trials in which subjects consecutively selected the same button as on the immediately preceding trial. Response time was defined as the latency from button onset to the subsequent button press on the second trial. Key predictors were condition (appearance vs. no-appearance), button type (low vs. intermediate in Experiment 1; high vs. intermediate in Experiment 2), and their interaction. To account for potential confounds, we included session number (continuous, 1–4), optional-choice trial number (continuous), and subject age (continuous) as control variables. As in the binomial choice model, sex was included as a fixed effect in Experiment 1 but not in Experiment 2. We included subject ID as a random intercept. We assessed the significance of the full model by comparing it with a null model that contained only the control variables and the random intercept, using a likelihood-ratio test via the *anova* function. When the interaction was significant, we conducted Tukey’s pairwise comparisons with the “emmeans” package, comparing button types within each condition while holding continuous covariates at their sample means and categorical covariates at their observed proportions.
