# Supplementary material for: Intrinsic recreation of moderately uncertain events in macaques
Source: iScience. 2026 Apr 18;29(5):115820. doi: 10.1016/j.isci.2026.115820 (PMC13156601; doi:10.1016/j.isci.2026.115820)

iScience, Volume *29*

Supplemental information

Intrinsic recreation of moderately uncertain events in macaques

Sakumi Iki, Haruhiko Iwaoki, Yuko Hattori, and Ikuma Adachi

Table S1. Raw choice proportions for each button type by condition in each experiment, related to Figures 2 and 3

Experiment	Condition	Button Type	Choice proportion (%)
**Experiment 1**	appearance	intermediate-noise	54.9
		low-noise	45.1
	no-appearance	intermediate-noise	48.6
		low-noise	51.4
**Experiment 2**	appearance	intermediate-noise	55.3
		high-noise	44.7
	no-appearance	intermediate-noise	39.6
		high-noise	60.4

Table S2. Details of the model comparison, related to Figures 2 and 3

Model		AIC	logLik	χ^2	df	p-value
**Experiment 1: Button choice**						
Null	BT ~ SN + TN + LB + S + A + (1|ID)	1120	−553.2			
Full	BT ~ C + SN + TN + LB + S + A + (1|ID)	1117	−550.4	5.51	1	**0.0189***
**Experiment 1: Response time**						
Null	RT ~ SN + TN + S + A + (1|ID)	10021	−5004			
Full	RT ~ BT*C + SN + TN + S + A + (1|ID)	10005	−4993	21.90	3	**<0.0001******
**Experiment 2: Button choice**						
Null	BT ~ SN + TN + LB + A + (1|ID)	506.0	−247.0			
Full	BT ~ C + SN + TN + LB + A + (1|ID)	502.9	−244.4	5.09	1	**0.0241***
**Experiment 2: Response time**						
Null	RT ~ SN + TN + A + (1|ID)	5554	−2771			
Full	RT ~ BT*C + SN + TN + A + (1|ID)	5537	−2760	22.62	3	**<0.0001******

BT: button type; RT: response time; SN: session number; TN: trial number; LB: last button type; S: sex; A: age; ID: subject ID; C: condition. * $p < 0.05$; **** $p < 0.0001$.

Table S3. Factors affecting button choice in Experiment 1, related to Figure 2

	**β**	**SE**	**z-value**	**p-value**
Intercept	−2.089	2.153	−0.970	0.332
Condition (no-appearance)	−0.387	0.166	−2.336	**0.019***
Trial number	0.009	0.005	1.882	0.060
Last button type (low-noise)	−0.312	0.152	−2.059	**0.040***
Session number	0.028	0.075	0.374	0.709
Sex (male)	0.389	0.720	0.539	0.590
Age	0.142	0.130	1.091	0.275

Sample size: N = 870 button choices. Subject ID was included as a random intercept. * $p < 0.05$.

Table S4. Supplementary GLMM restricted to the first and last five optional-choice trials within the appearance condition in Experiment 1, related to Figure 2

	β	SE	z-value	p-value
Intercept	0.572	2.132	0.268	0.788
Sub-block (last)	−0.530	0.487	−1.088	0.277
Last button type (low-noise)	−0.774	0.532	−1.455	0.146
Sex (male)	0.483	0.688	0.702	0.483
Age	−0.019	0.123	−0.148	0.882

Sample size: N = 80 button choices. Subject ID was included as a random intercept.

Table S5. Supplementary GLMM restricted to the no-appearance condition in Experiment 1, related to Figure 2

	β	SE	z-value	p-value
Intercept	−0.691	1.474	−0.469	0.639
Color/Side (yellow/left)	−1.392	0.425	−3.277	**0.001****
Trial number	0.011	0.006	1.971	**0.049***
Last button color/side (yellow/left)	0.036	0.202	0.179	0.858
Session number	−0.030	0.214	−0.142	0.887
Sex (male)	−0.677	0.637	−1.063	0.288
Age	0.068	0.084	0.817	0.414

Sample size: N = 475 button choices. Subject ID was included as a random intercept. * $p < 0.05$, ** $p < 0.01$.

Table S6. Supplementary GLMM restricted to the appearance condition in Experiment 1, related to Figure 2

	**β**	**SE**	**z-value**	**p-value**
Intercept	0.479	2.243	0.213	0.831
Color/Side (yellow/left)	−1.771	0.681	−2.602	**0.009****
Trial number	0.011	0.008	1.301	0.193
Last button color/side (yellow/left)	−0.367	0.247	−1.487	0.137
Session number	0.068	0.282	0.243	0.808
Sex (male)	0.020	0.716	0.028	0.978
Age	0.000	0.127	0.003	0.998

Sample size: N = 395 button choices. Subject ID was included as a random intercept. ** $p < 0.01$.

Table S7. Factors affecting response time in Experiment 1, related to Figure 2

	**β**	**SE**	**z-value**	**p-value**
Intercept	6.482	1.615	4.014	**<0.0001****
Button type (low-noise)	0.741	0.179	4.145	**<0.0001****
Condition (no-appearance)	0.025	0.146	0.169	0.8662
Trial number	0.024	0.004	6.162	**<0.0001****
Session number	0.367	0.059	6.217	**<0.0001****
Sex (male)	−0.094	0.538	−0.175	0.8612
Age	0.068	0.097	0.698	0.4852
Button type (low-noise) × Condition (no-appearance)	−0.669	0.216	−3.093	**0.0020**

Sample size: N = 512 consecutive same-button selections. Subject ID was included as a random intercept. ** $p < 0.01$; **** $p < 0.0001$.

Table S8. Tukey-adjusted post hoc pairwise comparisons of response time for each button type within each condition in Experiment 1, related to Figure 2

	ratio	SE	z-ratio	p-value
**Condition = Appearance:**				
Intermediate-noise / Low-noise	0.477	0.085	−4.145	**<0.0001********
**Condition = No-appearance:**				
Intermediate-noise / Low-noise	0.931	0.147	−0.451	0.6520

For the Tukey tests, the “emmeans” function from the R package *emmeans* was used. Estimated marginal means were computed with continuous covariates fixed at their sample means and categorical covariates averaged over their observed proportions. **** $p < 0.0001$.

Table S9. Factors affecting button choice in Experiment 2, related to Figure 3

	β	SE	z-value	p-value
Intercept	0.844	1.852	0.456	0.6485
Condition (no-appearance)	−0.543	0.242	−2.248	**0.0245***
Trial number	−0.016	0.010	−1.652	0.0985
Last button type (intermediate-noise)	0.438	0.234	1.869	0.0616
Session number	0.166	0.106	1.562	0.1183
Age	−0.053	0.115	−0.461	0.6445

Sample size: N = 404 button choices. Subject ID was included as a random intercept. * $p < 0.05$.

Table S10. Supplementary GLMM restricted to the first and last five optional-choice trials within the appearance condition in Experiment 2, related to Figure 3

	β	SE	z-value	p-value
Intercept	−0.411	2.209	−0.186	0.852
Sub-block (last)	−0.142	0.666	−0.213	0.831
Last button type (intermediate-noise)	0.393	0.766	0.513	0.608
Age	0.033	0.141	0.237	0.813

Sample size: N = 45 button choices. Subject ID was included as a random intercept.

Table S11. Supplementary GLMM restricted to the no-appearance condition in Experiment 2, related to Figure 3

	**β**	**SE**	**z-value**	**p-value**
Intercept	−0.055	1.402	−0.039	0.969
Color/Side (purple/right)	0.906	0.457	1.981	**0.048***
Trial number	−0.035	0.018	−1.924	0.054
Last button color/side (purple/right)	−0.226	0.357	−0.633	0.527
Session number	0.529	0.333	1.587	0.113
Age	−0.052	0.080	−0.655	0.513

Sample size: N = 187 button choices. Subject ID was included as a random intercept. * $p < 0.05$.

Table S12. Supplementary GLMM restricted to the appearance condition in Experiment 2, related to Figure 3

	β	SE	z-value	p-value
Intercept	0.661	2.004	0.330	0.742
Color/Side (purple/right)	1.175	0.728	1.614	0.107
Trial number	−0.009	0.012	−0.720	0.471
Last button color/side (purple/right)	−0.519	0.318	−1.633	0.103
Session number	0.285	0.331	0.862	0.389
Age	−0.035	0.118	−0.297	0.766

Sample size: N = 217 button choices. Subject ID was included as a random intercept.

Table S13. Factors affecting response time in Experiment 2, related to Figure 3

	**β**	**SE**	**z-value**	**p-value**
Intercept	7.670	2.760	2.779	**0.0055****
Button type (intermediate-noise)	−1.083	0.329	−3.290	**0.0010****
Condition (no-appearance)	0.255	0.288	0.886	0.3754
Trial number	0.056	0.010	5.606	**<0.0001******
Session number	0.541	0.110	4.904	**<0.0001******
Age	0.081	0.171	0.472	0.6369
Button type (intermediate-noise) × Condition (no-appearance)	1.216	0.452	2.690	**0.0071****

Sample size: N = 251 consecutive same-button selections. Subject ID was included as a random intercept. ** $p < 0.01$; **** $p < 0.0001$.

Table S14. Tukey-adjusted post hoc pairwise comparisons of response time for each button type within each condition in Experiment 2, related to Figure 3

	ratio	SE	z-ratio	p-value
**Condition = Appearance:**				
High-noise / Intermediate-noise	2.952	0.971	3.290	**0.0010****
**Condition = No-appearance:**				
High-noise / Intermediate-noise	0.875	0.352	−0.331	0.7404

For the Tukey tests, the "emmeans" function from the R package *emmeans* was used. Estimated marginal means were computed with continuous covariates fixed at their sample means and categorical covariates averaged over their observed proportions. ** $p < 0.01$.

Figure S1. Probability of continued participation in the game as a function of the inter-press interval in Experiment 1, estimated using Kaplan–Meier survival analysis, related to STAR Methods. τ denotes the earliest time point at which the probability of continuation is ≤ 5%. The red shaded region indicates the confidence interval.

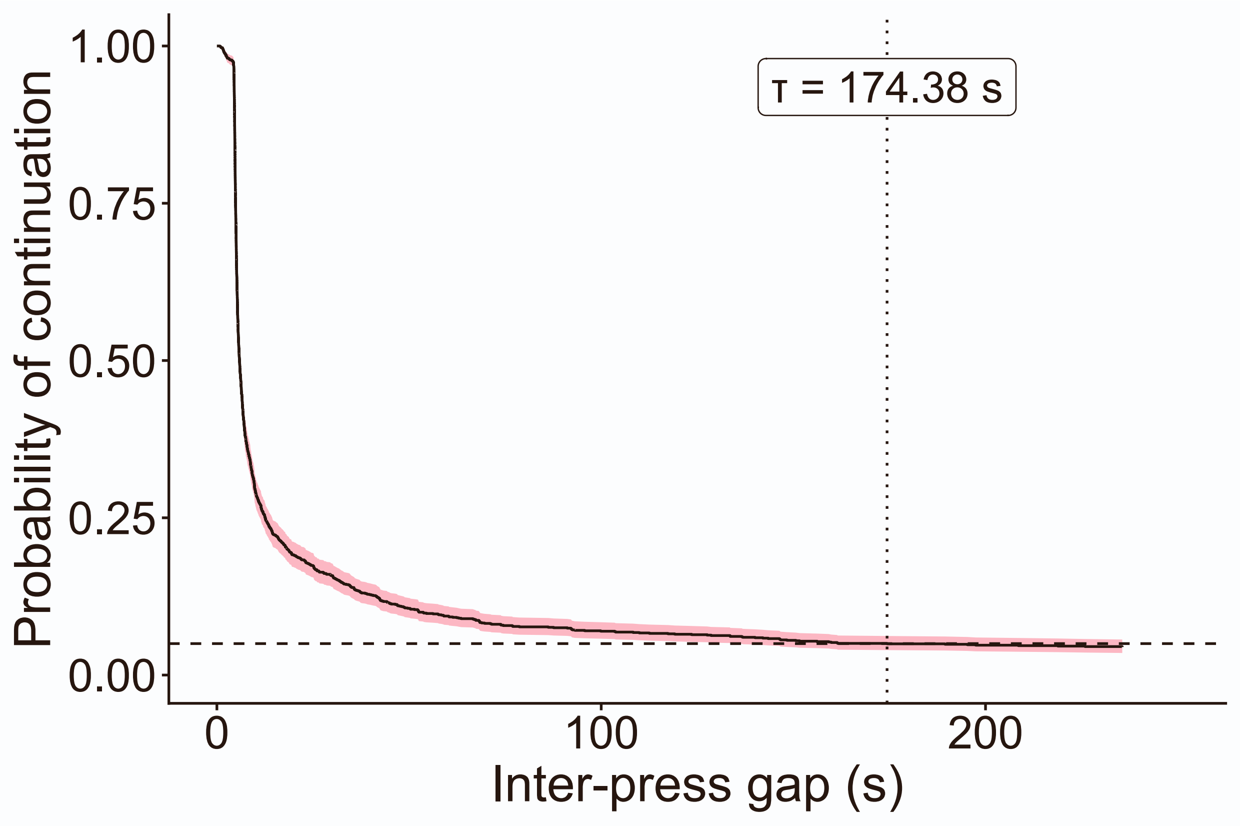

Figure S2. Predicted response time for trials in Experiment 1 in which subjects consecutively selected the same button, as a function of (A) optional-choice trial number and (B) session number, related to Figure 2. Estimated marginal means were computed with continuous covariates fixed at their sample means and categorical covariates averaged over their observed proportions. The red shaded region indicates the confidence interval. In panel (**A**), points are subject-level means computed in 10-trial bins; in panel (**B**), points are subject-level means for each session. Point size is proportional to the number of observations.

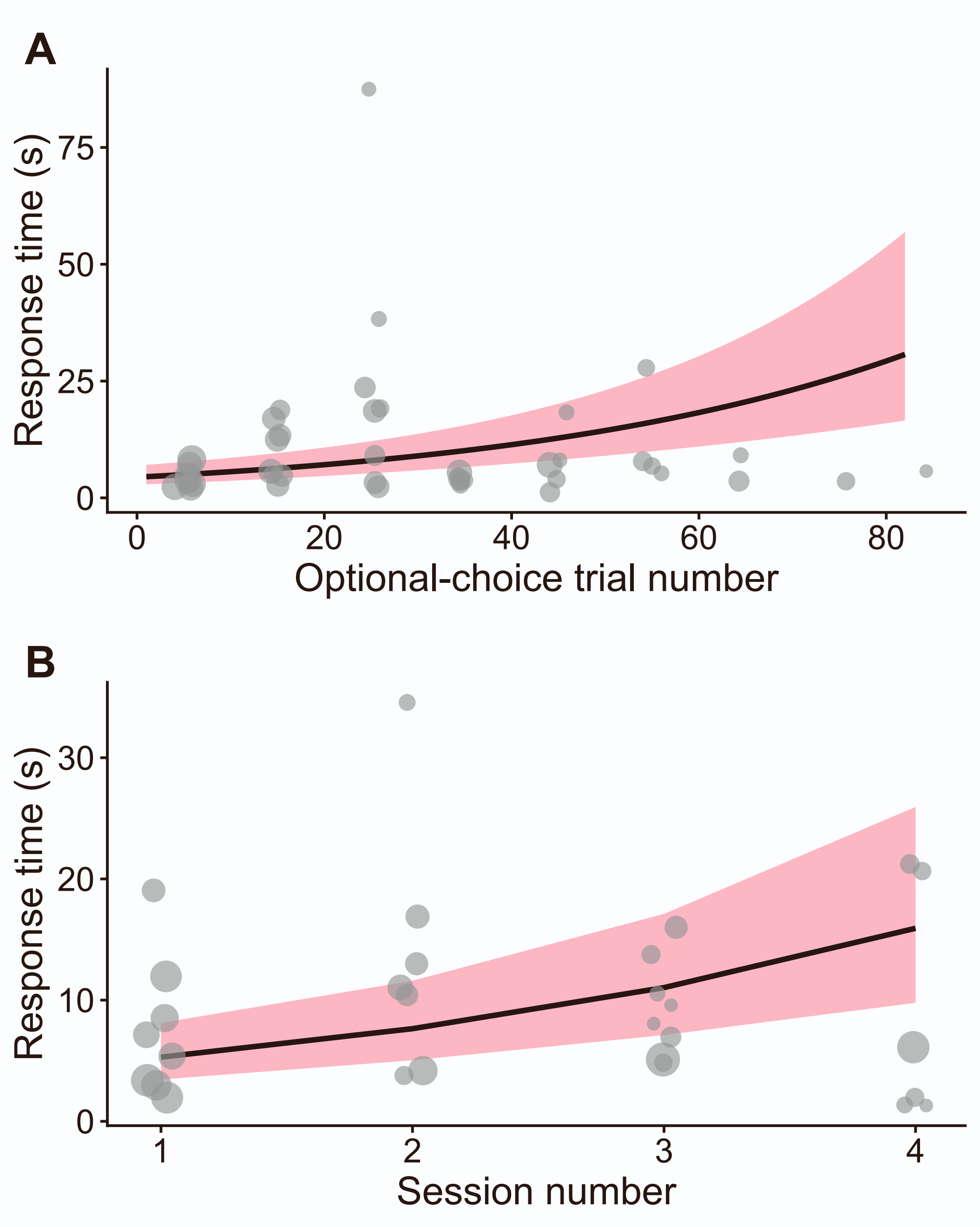

Figure S3. Probability of continued participation in the game as a function of the inter-press interval in Experiment 2, estimated using Kaplan–Meier survival analysis, related to STAR Methods. τ denotes the earliest time point at which the probability of continuation is ≤ 5%. The red shaded region indicates the confidence interval.

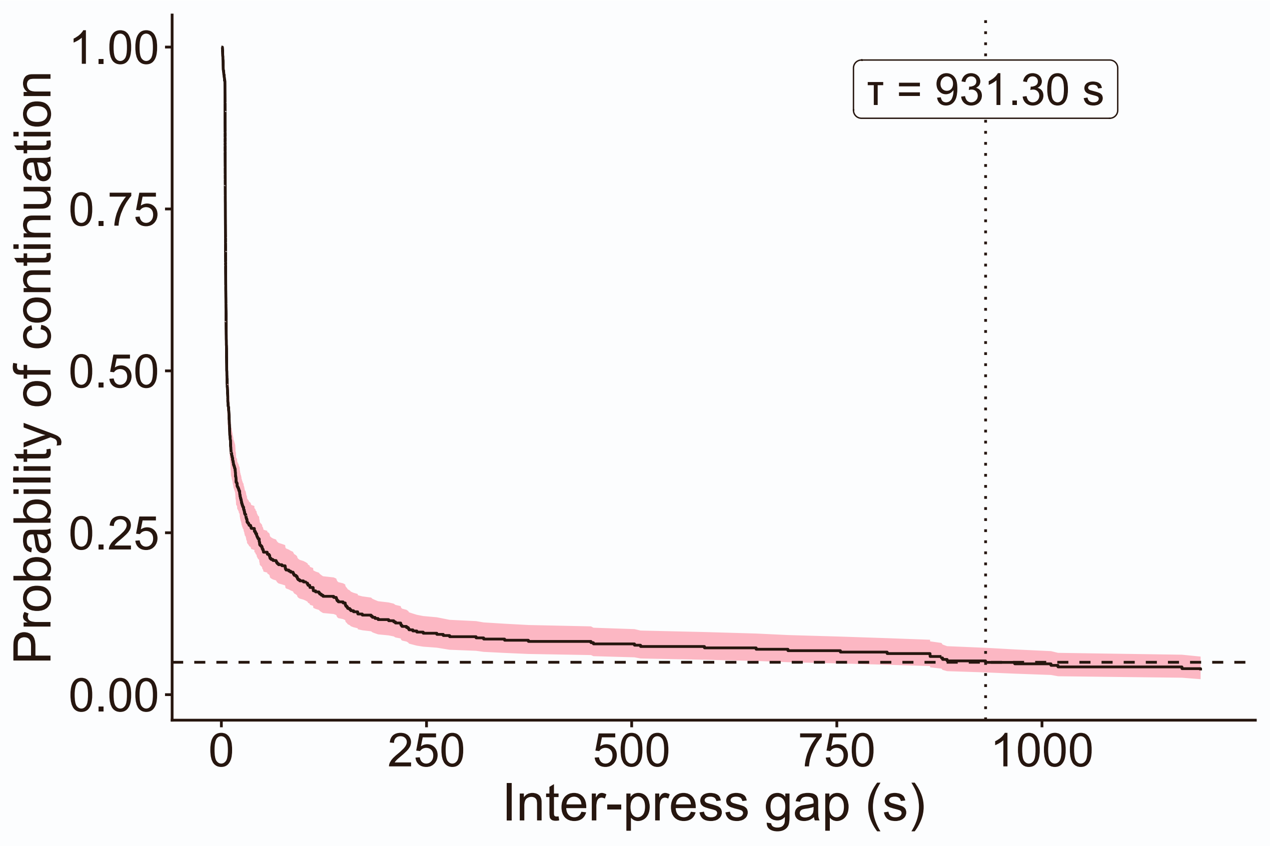

Figure S4. Predicted response time for trials in Experiment 2 in which subjects consecutively selected the same button, as a function of (A) optional-choice trial number and (B) session number, related to Figure 3. Estimated marginal means were computed with continuous covariates fixed at their sample means and categorical covariates averaged over their observed proportions. The red shaded region indicates the confidence interval. In panel (**A**), points are subject-level means computed in 10-trial bins; in panel (**B**), points are subject-level means for each session. Point size is proportional to the number of observations.

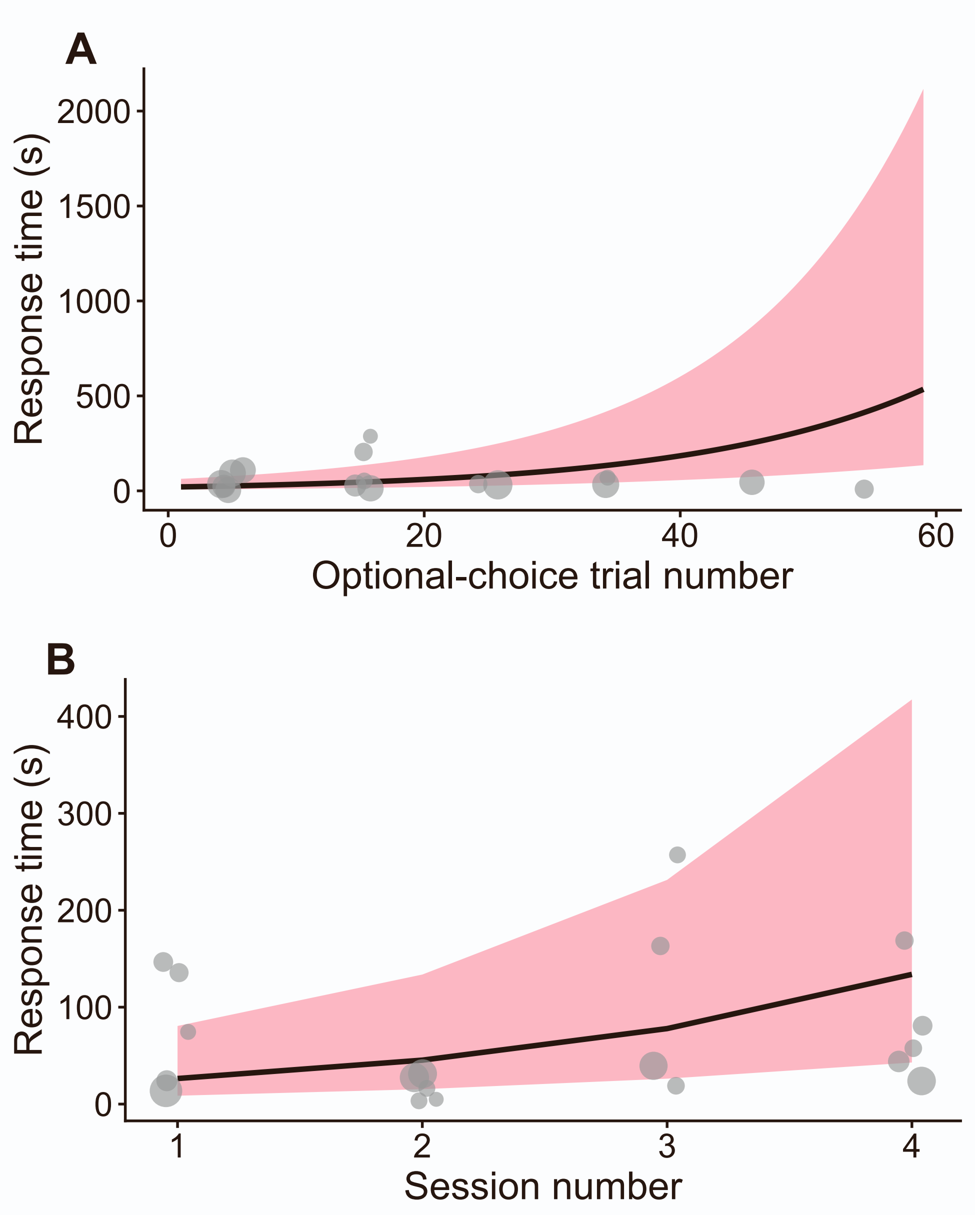

Supplement: Document S1. Figures S1–S4 and Tables S1–S14 [file mmc1.pdf]
